# Traumatic Phthisis

**Published:** 1874-03

**Authors:** C. F. Bevan

**Affiliations:** Demonstrator of Anatomy and Lecturer on Osteology in the College of Physicians and Surgeons, Baltimore.


### SELECTED ARTICLES.

---

### ARTICLE III.

### *Traumatic Phthisis.*

BY C. F. BEVAN, M. D.

Demonstrator of Anatomy and Lecturer on Osteology in the College of Physicians and Surgeons, Baltimore.

Within the last few years, the general pathology and doctrines respecting Phthisis Pulmonalis have undergone many and marked changes. The views of Lennec, "that *every* form of phthisis pulmonalis is caused by a *specific new growth*, and that the cavities in the lung take their origin *alone* in softening and the evacuation of this growth," are no longer considered tenable. They have been too frequently refuted to need comment or explanation here. Still, though we do not accept the *specific new growth* theory as a whole, we are far from rejecting it entirely. That miliary tubercles are sometimes found, is acknowledged ; yet these miliary growths form by far the smallest percentage of all consumptive cases. Nor are we willing to admit that "almost all the alleged miliary tubercles of the lungs are bronchitic, peribronchitic and pneumonic deposits," even when endorsed by Virchow. It is by no means an unfrequent occurrence to find both the yellow caseous deposits, and the small, granular, miliary growths or tubercles associated in the same lung, as well as scattered throughout the system at large.

The beautiful views of Niemeyer, assigning the majority of consumptive cases "to consolidation and destruction of the lung from pneumonic processes, especially chronic catarrhal pneumonia," are now almost universally received. The morbid changes resulting from the pneumonic processes, viz: consolidation of the lung tissue, in a mechanical form, simply by an increased proliferation of young, indifferent, undeveloped round cells filling up the alveoli and undergoing cheesy metamorphosis, account fully for the pathological lesions found. But whether we accept the specific new growth theory, or that the alleged miliary tubercles are bronchitic, peribronchitic, or pneumonic deposits; or whether we accept the views of Niemeyer, different as they are in their pathology, yet in one respect at least they all agree, viz: that *all* cases of phthisis pulmonalis commence with lobular induration, and end in disintegration with cavernous formation.

That phthisis may be produced artificially by inoculation, the experiments of Burdon, Sanderson, Wilson, Fox, Cohnheim, Waldenberg, Villemin, and others, fully demonstrate. These experimenters produced phthisis, usually by the introduction or inoculation of matter from diseased glands into the pleural or peritonial cavities; the introduction of setons and a variety of other bodies, harmless in themselves, was followed by the same results. Their observations were made upon animals, especially the guinea pig, but the same results would have ensued if practiced upon the human species, as the following case will prove.

*Case.*—John H. Jones, æt. 45, color black, died Nov. 11th, 1873. Twelve hours after death the body was brought to the dissecting room of the College of Physicians and Surgeons. The subject was of large size, though greatly emaciated. On examination, a small fistulous orifice was found in the right axillary space; the probe was passed in through the orifice and entered the pleural cavity. As the muscles were removed and the ribs exposed, a most extensive necrosis and caries was revealed, about four (4) inches vertically by two (2) inches horizontally, involving the second, third, fourth, fifth and sixth ribs. The inferior border of the second, and external surface of the third ribs were carious; while the fourth and fifth ribs exhibited marks of previous violence to such an extent as to destroy a portion of them; the superior border of the sixth rib was also carious. The diseased parts were situated just under the Serratus Magnus muscle, which had been partly detached, by infiltration of pus, at its lower portion. The feature of greatest interest was connected with the fourth and fifth ribs, in which was found an opening, one inch square, communicating directly with the lung substance. From the examination of the bones, it was evident that a fracture of the ribs, attended by much contusion, had previously occurred. The lungs were next subjected to a rigid examination; the pleura, on the right side, was firmly adherent both to lung and chest walls, whilst on the left side the adhesions were slight; no effusion on either side. Both lungs were densely consolidated, and exhibited various stages of degeneration; indurated masses, cheesy deposits, and cavities. On the right side, at the point corresponding to the opening in the bones, a circumscribed pleuritis had evidently existed, and a large cavity communicating with and discharging through the osseous opening was found. The right lung was more diseased than the left. The glandular system showed great enlargement, especially the parotids and mesenteric. Brain not examined.

So remarkable were the lesions, that a careful investigation was instituted to obtain the history of the subject, which, though extremely arduous and difficult, fully repaid the labor. It was found that the man had enjoyed remarkably good health, free from all indications either of pulmonary or syphilitic trouble, up to the last three years since he received an injury by the explosion of a steam boiler. After the injury he lost his health and strength; was frequently sick, with pulmonary trouble; became unable to attend to his regular work, supporting himself by odd jobs, &c. His family history was good.

The injury received was undoubtedly a fracture of the fourth and fifth ribs, and the broken end had pierced the lung substance, causing the circumscribed pleuritis. The cavity communicating with the external orifice was evidently an abscess produced by the fragments of bone, and, from the great inflammation thereby developed, as well as from the pressure, the aforementioned ribs had become carious. The case was certainly one of phthisis pulmonalis; and from the absence of all hereditary taint, from the absence of all suspicion of previous sickness, as well as the character of the lesions found, should be classed as *Traumatic.* The broken bone irritating the lung had developed the abscess, congestion and pneumonitis had resulted, only to be followed by the pathological change common to all consumptive cases, viz: lopular induration, terminating in disintegration and cavernous formation.

Now from this case it can at once be seen, that the law governing the artificial production of phthisis in the animal, applies with equal force to man; for we may consider the broken bone, irritating the lung, as the source of the evil. The same case also furnishes another illustration of Niemeyer's theory, since pneumonic changes had undoubtedly produced the lesions found.

Will not this case account for the large number of consumptives found at our large manufacturing establishments —among iron workers, among stone cutters and coal miners, who work in an atmosphere filled with dust, small pieces of iron, stone and coal? Perhaps it may be argued that but little reliance can be placed on this case, since it was a *subject*, and since tubercles are frequently found in cases who have died from other causes. True! Nothing is more common than to find tubercles associated with other lesions, in the dead house; yet the family history of the case under discussion; the singular immunity from sickness prior to the date of injury; the entire adequacy of the injury found to produce and account for the lesions; the radiating of the tuberculous matter from the point of injury, as a focus, to the whole system; and, finally, the length of time consumed by it, (three years)—are all points of evidence which properly considered, contraindicate a tubercular deposit prior to the injury, or death from causes other than Traumatic Phthisis.—*Physician and Surgeon.*

